# Transcription factor E2F1 promotes EMT by regulating ZEB2 in small cell lung cancer

**DOI:** 10.1186/s12885-017-3701-y

**Published:** 2017-11-07

**Authors:** Tingting Wang, Xufang Chen, Weiwei Qiao, Lijun Kong, Daqing Sun, Zunling Li

**Affiliations:** 1 Department of Biochemistry and Molecular Biology, Binzhou Medical University, Yantai, 264003 China; 2 Tianjin Medical University General Hospital, Tianjin, 300052 China; 3 Oncology Department, Yantai Affiliated Hospital of Binzhou Medical University, Yantai, 264199 China; 4 Department of Diagnostics, Binzhou Medical University, Yantai, 264003 China

**Keywords:** Epithelial-mesenchymal transition, E2F1, Small cell lung cancer, ZEB2

## Abstract

**Background:**

Epithelial-mesenchymal transition (EMT) is an early event in tumour invasion and metastasis, and widespread and distant metastasis at early stages is the typical biological behaviour in small cell lung cancer (SCLC). Our previous reports showed that high expression of the transcription factor E2F1 was involved in the invasion and metastasis of SCLC, but the role of E2F1 in the process of EMT in SCLC is unknown.

**Methods:**

Immunohistochemistry was performed to evaluate the expressions of EMT related markers. Immunofluorescence was used to detect the expressions of cytoskeletal proteins and EMT related markers when E2F1 was silenced in SCLC cell lines. Adenovirus containing shRNA against E2F1 was used to knock down the E2F1 expression, and the dual luciferase reporter system was employed to clarify the regulatory relationship between E2F1 and ZEB2.

**Results:**

In this study, we observed the remodelling of cytoskeletal proteins when E2F1 was silenced in SCLC cell lines, indicating that E2F1 was involved in the EMT in SCLC. Depletion of E2F1 promoted the expression of epithelial markers (CDH1 and CTNNB1) and inhibited the expression of mesenchymal markers (VIM and CDH2) in SCLC cell lines, verifying that E2F1 promotes EMT occurrence. Next, the mechanism by which E2F1 promoted EMT was explored. Among the CDH1 related inhibitory transcriptional regulators ZEB1, ZEB2, SNAI1 and SNAI2, the expression of ZEB2 was the highest in SCLC tissue samples and was highly consistent with E2F1 expression. ChIP-seq data and dual luciferase reporter system analysis confirmed that E2F1 could regulate *ZEB2* gene expression.

**Conclusion:**

Our data supports that E2F1 promotes EMT by regulating *ZEB2* gene expression in SCLC.

**Electronic supplementary material:**

The online version of this article (10.1186/s12885-017-3701-y) contains supplementary material, which is available to authorized users.

## Background

The cancer statistics for China in 2015 showed that there were 733,330 new cases of lung cancer (509,300 cases in males and 224,000 in females), and 610,200 deaths (432,400 males and 177,800 females) due to lung cancer. The incidence and mortality rate for lung cancer ranked first out of all tumours [1]. Small cell lung cancer (SCLC) is one of the most malignant tumours and accounts for 20–25% of all lung cancers. With the aggravation of environmental pollution, the incidence of SCLC is increasing year by year, and the five-year survival rate remains at approximately 10% [2]. One of the typical biological behaviours of SCLC is widespread, distant metastases at early stages [3]. In addition, *RB1* (RB transcriptional corepressor 1) gene loss, which is a typical genetic characteristic of SCLC, leads to the deregulation of E2F1 [4]. Our previous research showed that E2F1 is highly expressed in SCLC [2], indicating that E2F1 plays a role in SCLC.

E2F1 is a transcription factor that is involved in the cell cycle, proliferation, apoptosis and differentiation [5]. Recent reports showed that E2F1 took part in tumour invasion and metastasis by regulating thrombospondin 1 [6], PDGFR [7] and VEGFR [8]. Our previous research showed that E2F1 was also involved in invasion and metastasis by controlling MMP-9, MMP-16 and ADAM-12 [2, 3, 9]. We also observed changes in cell morphology when E2F1 was silenced, indicating that E2F1 may regulate the epithelial-mesenchymal transition (EMT).

EMT is an early event in the process of tumour invasion and metastasis [10]. E2F1 can suppress the Wnt/β-catenin signalling pathway by regulating ICAT [11] and GSK-3 [12] in colorectal cancer and can also drive EMT by inducing miR-224/452 in malignant melanoma [13]. These results showed that E2F1 is closely associated with EMT, but the detailed mechanism of EMT regulation by E2F1 in SCLC is unknown.

In this study, we analysed the expression pattern of EMT-related proteins in SCLC tissue samples. We then examined the changes in cell morphology and cytoskeleton remodelling processes when E2F1 was knocked down by shRNA in SCLC cells. In these cells, epithelial markers were significantly increased and mesenchymal markers were significantly decreased. ChIP-seq and dual-luciferase reporter experiments indicated that E2F1 directly regulated the expression of *ZEB2* by binding to its promoter. Our results suggest that E2F1 promotes EMT by regulating *ZEB2* in SCLC.

## Methods

### Patients and cell lines

Sixty SCLC biopsy tissue samples before treatment were obtained from the affiliated hospital of Binzhou Medical University from January 2014 to January 2015. All patients signed informed consent forms before providing tissue samples. This research was approved by the Medical Ethics Committee of Binzhou Medical University (No. 2013027). This study was performed according to the Declaration of Helsinki and to the relevant ethical guidelines for research on humans. The basic patient information is listed in Table 1. Human SCLC cell lines H446 (TCHu196, Chinese Academy of Sciences cell bank) and H1688 (TCHu154, Chinese Academy of Sciences cell bank) were stored in our lab. All cells were cultured in RPMI 1640 media (Gibco, Cat:89,984) with 10% FBS (Gibco, Cat: 26,140,079) and 100 U/ml penicillin and 100 μg/ml streptomycin.

**Table 1:** Clinicopathologic features for patients suffering SCLC (*n* = 60)

Variables		N (%)	Statistical analysis		
			HR	95%CI	*P*
Age	<60	16(26.67)	1	0.297–1.558	0.362
	≥ 60	44(73.33)	0.681		
Gender	Male	55(91.67)	1	0.900–8.409	0.076
	Female	5(8.33)	2.752		
Smoking	Non-smoker	4(6.67)	1	0.603–4.697	0.321
	Smoker	56(93.33)	1.682		
Tumor size	< 4 cm	24(40.00)	1	0.344–1.397	0.306
	≥ 4 cm	36(60.00)	0.693		
Clinical stage	Limited disease	8(13.33)	1	0.072–0.831	**0.024**
	Extensive disease	52(86.67)	0.224		

*represents *p*<0.05

### Immunohistochemistry

All tissue sections were dewaxed in dimethylbenzene and rehydrated in an alcohol gradient ranging from 100% to 75%. Antigen retrieval was performed by placing samples in an EDTA antigen repair solution (ph = 9.0). Sections were then washed three times with PBS buffer (ph = 7.0). Next, the sections were incubated with a primary antibody at 4 °C overnight. The antibodies were used at the following dilutions: 1:100 for VIM (Cell signalling technology, Cat:5741), 1:150 for CDH2 (Cell signalling technology,Cat:13,116), 1:250 for CLDN1 (Cell signalling technology, Cat:13,255), 1:100 for CTNNB1 (Cell signalling technology, Cat:8480), 1:400 for CDH1 (Cell signalling technology, Cat:3195), 1:50 for E2F1 (Santa Cruz, Cat:sc-251), 1:200 for ZEB2 (Santa Cruz, Cat:271,984), ZEB1 (Cell signalling technology, Cat:3396), SNAI1 (Cell signalling technology, Cat:3879), and SNAI2 (Cell signalling technology, Cat:9585). The sections were washed three times with PBS and incubated with HRP-conjugated secondary antibodies for 40 min at 37 °C. Then, the DAB (diaminobenzidine) reaction, hematoxylin staining, differentiation with hydrochloric acid alcohol, dehydration and transparency steps were conducted in turn. All images were captured by Leica Microsystems CMS (DFC365 FX). All staining was scored according to our previous reports [2, 3]. In brief, the staining was quantified using a 4-value intensity score: 0 as negative; 1+ as weak; 2+ as moderate, 3+ as strong, and the percentage (0–100) of the extent of reactivity. A final score was obtained by multiplying the intensity and reactivity extension values (range, 0–300) [14].

### H446 cells in which E2F1 was stably knocked down by adenovirus containing E2F1 specific shRNA were constructed

Adenovirus (1 × 10^9^ titers) containing shRNA against E2F1 was provided by Gene Pharma Company. H446 cells were conventionally cultured in six-well plates. At a confluence of approximately 70%–80%, 1 × 10^6^ virus titers were added and mixed gently. After 12 h, the completed medium was added to cells and the previous medium was removed. After 72 h, puromycin (5 μg/ml) was added to the media. After 5 days, all cells were digested, diluted, and cultured in a 96-well plate to form monoclonal colonies. The medium containing puromycin (1 μg/ml) was changed every 3 days until the confluence was approximately 90%–100%. Cells were then digested and inoculated into 48-well, 24-well and 6-well plates. These cells were named H446-E2F1sh.

### siRNA and transfection

siRNAs targeting E2F1 was transfected into H1688 according to the methods stated in our previous report [2]. H1688 cells in which E2F1 was transiently silenced were named H1688-E2F1si.

### Real-time PCR.

Total RNA was extracted with RNAiso Plus (Takara, Cat: 9108), and cDNA was synthesized with the Prime Script RT reagent kit with gDNA Eraser (Takara, Cat: RR047A). Real-time PCR was performed according to the instructions provided for the SYBR Fast qPCR Mix (Takara, Cat: RR430A). The primers used are listed in additional file 1.

### Western blotting

All cells were lysed with RIPA lysis buffer containing a protease inhibitor cocktail (Sigma, Cat: S8820). The protein concentrations were measured, and 50 μg of protein was run on an SDS-PAGE gel and electrophoretically transferred onto NC membranes. The membranes were blocked with 5% fat-free milk and incubated overnight at 4 °C with primary antibodies, including VIM (1:1000), CDH2 (1:1000), CTNNB1 (1:1000), CDH1 (1:1000), E2F1 (1:500), ZEB2 (1:500), α-tubulin (1:1000), β-actin (1:1000) and GAPDH (1:2000). The membranes were then washed three times and incubated for 40 min with HRP-conjugated secondary antibodies. Protein bands were detected by the ECL system [2, 3].

### Immunofluorescence

Cells were placed on slides, washed three times with PBS buffer, and fixed with ice-cold methanol and acetone (1:1). Next, the slides were blocked for 30 min with goat serum, washed three times with PBS-TX (PBS containing 1% Tritonx-100), and incubated overnight at 4 °C with primary antibodies. The slides were then incubated with Rhodamine or FITC-labelled fluorescent secondary antibodies and DAPI. Laser scanning confocal microscope (leica-LM7000) was used to observe cell morphology and capture pictures.

### Construction of *ZEB2* luciferase reporter vector and activity analysis

Genomic DNA was extracted from H446 cells, and the *ZEB2* promoter was amplified by PCR and purified. The PCR fragment and pGL3-basic vector were digested with Nhe I and Bgl II enzymes, and T4 DNA ligase was used to ligate these two fragments together to construct the *ZEB2* promoter reporter vector. Luciferase activity analysis was performed according to methods described in our previous report [2].

### Statistical analysis

SPSS.17.0 statistical software and GraphPad Prism 7 was used. The results from the IHC experiments were analysed by a Chi Square test. The expression differences among target genes were analysed by a *t*-test. Multivariate survival analysis was performed by Cox’s regression.

## Results

### The expression of EMT markers in SCLC tissue samples

**Fig. 1:**
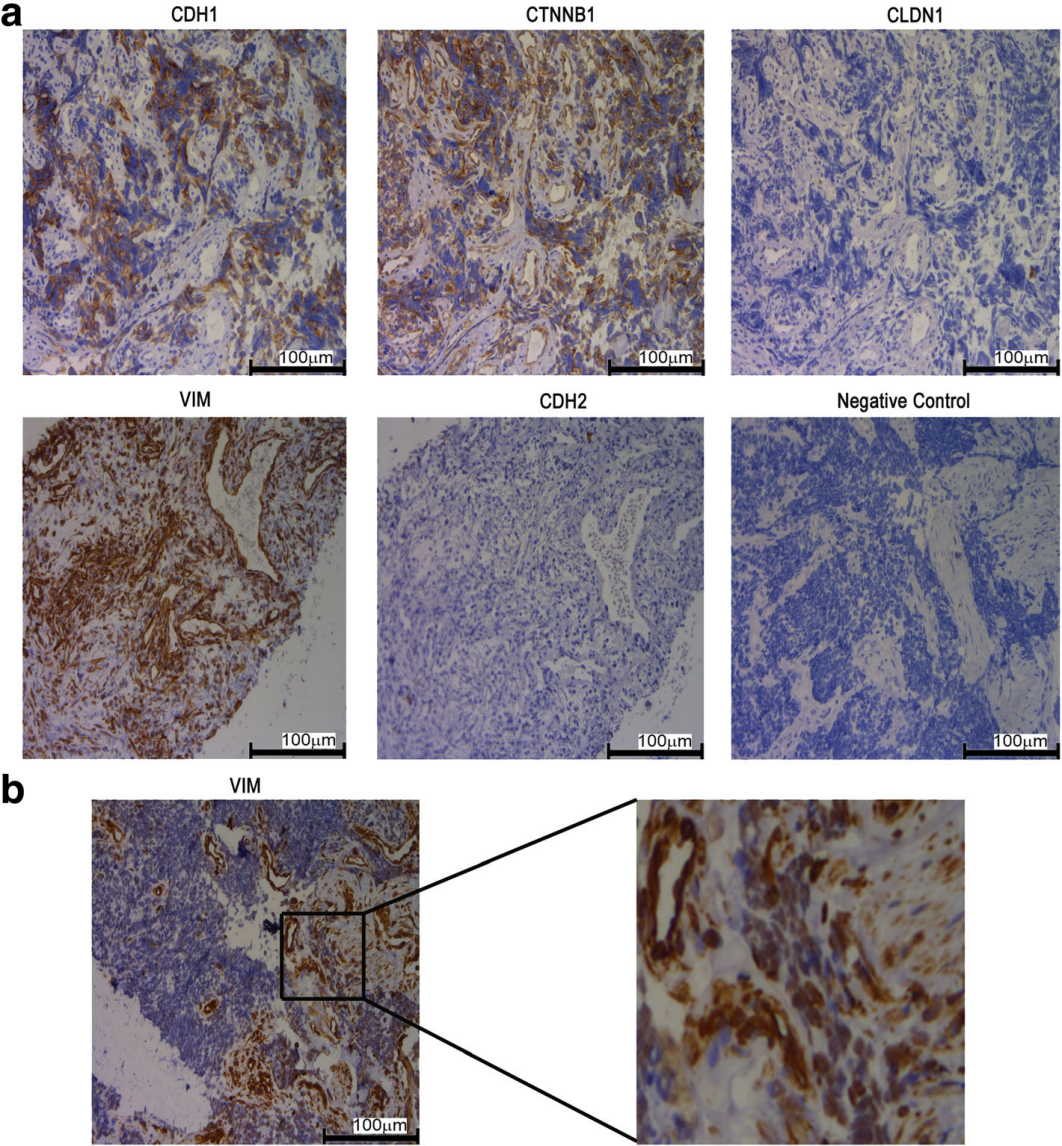
The immunohistochemical (IHC) staining of EMT related markers. **a**. EMT related markers, including CDH1, CTNNB1, CLDN1, VIM and CDH2, were detected by IHC in 60 SCLC tissue samples. **b** VIM expression was inconsistent in the same tissue sample. Representative image was presented, and the magnification is 200 X

Distant metastasis in early stages is a typical feature of SCLC, and the occurrence of EMT is considered to be an early event in the process of tumour invasion and metastasis. Therefore, it is important to determine the expression pattern of EMT markers in SCLC tissue. We examined CDH1 expression and found that it was highly related with CTNNB1 expression (*r* = 0.9985, (*p* < 0.001) and that both proteins were located on the cellular membrane and absent from the cytoplasm and nucleus. CTNNB1 and CDH1 were expressed in 90% (54/60) of small cell lung cancer cells (Fig. 1a), in which weak expression (score between 10 and 20) was 11.11%, moderate (score between 20 and 100) was 70.37% and strong (score between 100 and 300) was 18.52% (Additional file 2: Figure S1). This result was consistent with other reports [15–17]. CDH1 and CTNNB1 in bronchial epithelial cells as positive control were presented in Additional file 3: Figure S2. The Spearman analysis showed that CDH1, CTNNB1 were significantly relevant with tumour sizes (*p* = 0.03) and clinical stage (*p* < 0.001) not age (*p* = 0.461), gender (*p* = 0.335), smoking (*p* = 0.224). CTNNB1 has been previously observed in the nucleus when CDH1 was deregulated [18, 19]; however, we did not detect CTNNB1 in the nucleus (Fig. 1a, Additional file 2: Figure S1 and Additional file 3: Fig. S2), which was consistent with other reports [20]. This result supported that CTNNB1 did not transfer into the nucleus in SCLC samples. In additional, we analysed *CDH1* and *CTNNB1* expressions from the gene expression database of SCLC cell lines (https://sclccelllines.cancer.gov/) [21] (Additional file 4: Figure S3), and found that their expressions were not related in SCLC cell lines (*r* = 0.01671, *p* = 0.443). This told us that the expression pattern of *CDH1* and *CTNNB1* was different between SCLC tissues and SCLC cell lines.

CLDN1 and CDH2 were not detected in any SCLC samples (0/60) (Fig. 1a). VIM was observed in all stromal cells (Fig. 1a) and 25% (15/60) tumour cells (Fig. 1b), and its expression was significantly related with tumour differentiation (*p* < 0.001) [22].

Our previous paper reported that E2F1 was highly expressed and was an independent and adverse prognostic factor for SCLC. We also found that E2F1 could directly regulate the expressions of *RELA*, *MMPs* and *ADAM12* [2, 3, 9]. NF-κB, MMPs and ADAMs were found to be closely associated with EMT [23], indicating that E2F1 might promote EMT in SCLC.

### Depletion of E2F1-induced cell morphology changes in H446 and H1688 cells

**Fig. 2:**
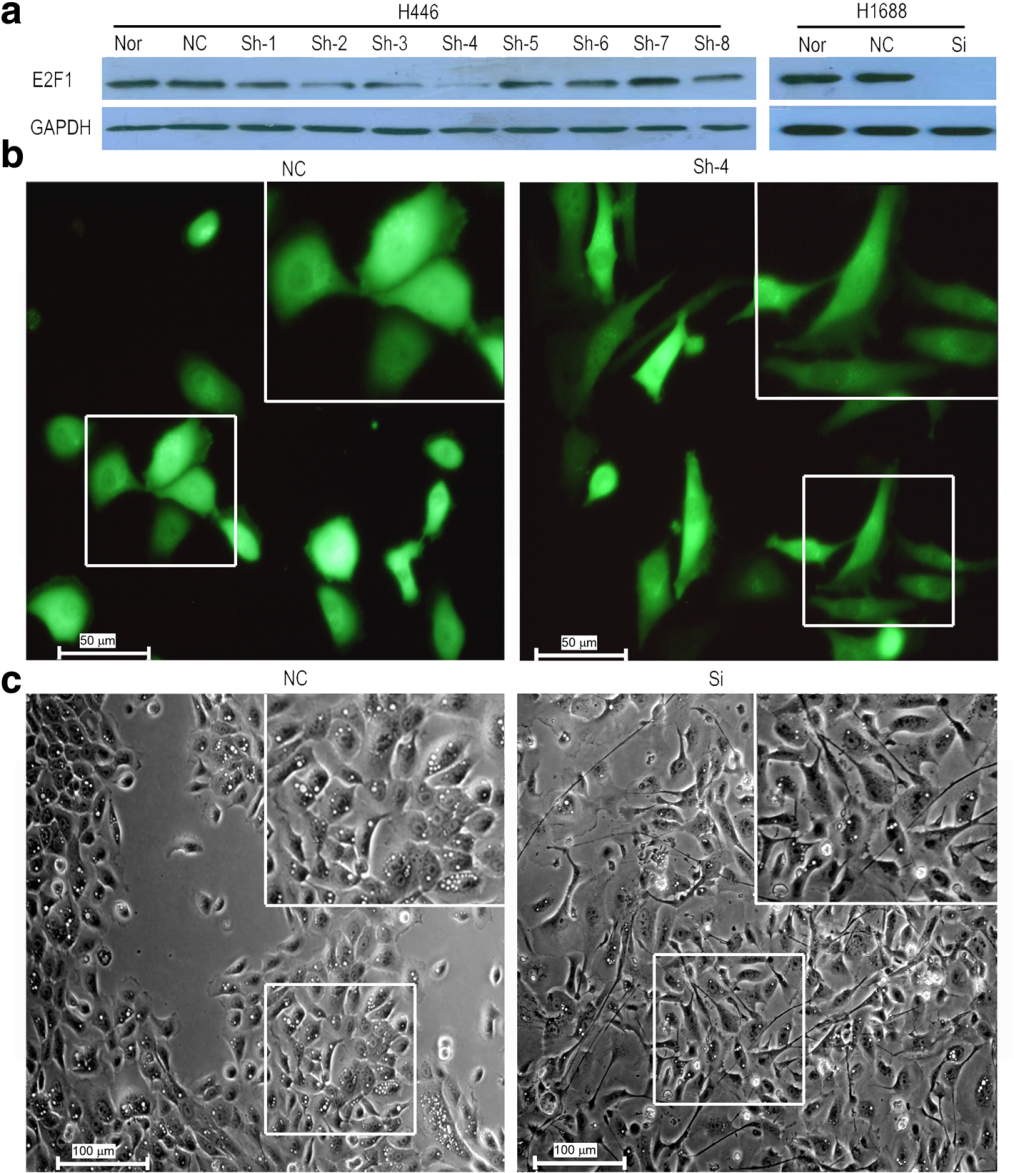
The cellular morphology was changed when *E2F1* was depleted in SCLC cells. **a** E2F1 protein was examined in 8 clones where *E2F1* was knocked down by shRNA in H446 cells (H446-E2F1sh) and transiently silenced by *E2F1* specific siRNA in H1688 cells (H1688-E2F1si). **b** Cellular morphology was changed from grain-shaped to slender and fibrous in clone-4 of H446-E2F1sh and H1688-E2F1si cells. Because shRNA vector contained eGFP, H446-E2F1sh cells were green. The magnification of H446 (panel **b**) was 400 X, and the magnification of H1688 (panel **c**) was 200 X

In our previous research, we found that the cellular morphology was changed when *E2F1* gene was silenced by specific siRNAs in SCLC. To further study cellular morphology, stable *E2F1* gene knockdown cells were generated using an adenovirus containing shRNA against *E2F1* in H446 cells (H446-E2F1sh cells). Eight clones were randomly tested for *E2F1* expression. *E2F1* was efficiently knocked down in clone-4 (Fig. 2a and Additional file 5: Figure S4), and clone-4 was then used to explore the relationship between E2F1 and EMT. The cellular morphology was significantly changed in clone-4 compared to negative control cells. When E2F1 was knocked down, cells changed from being grain-shaped to appearing slender and fibrous (Fig. 2b). The same change was observed when *E2F1* was silenced using siRNA against *E2F1* in H1688 cells (Fig. 2a, c and Additional file 5: Figure S4). Combined with our previous results [2, 3], we considered that E2F1 might regulate EMT to promote invasion and metastasis in SCLC.

### The expression of cytoskeletal proteins was changed when *E2F1* was knocked down.

**Fig. 3:**
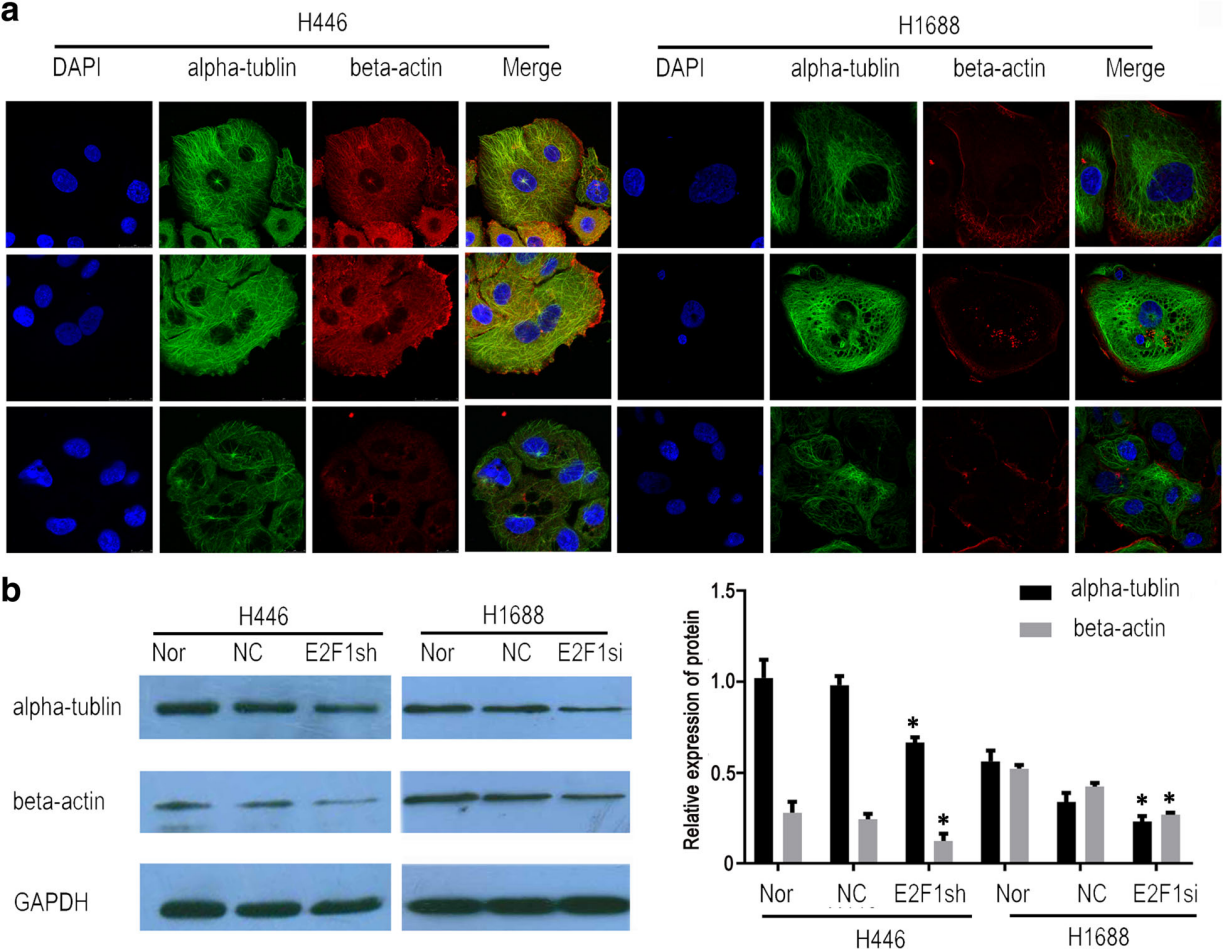
Depletion of *E2F1* influenced the expression of cellular cytoskeletal proteins. The expression levels of cellular cytoskeletal proteins including α-tublin and β-actin were decreased by immunofluorescence (**a**) and western blotting (**b**) in H446-E2F1sh and H1688-E2F1si cells. The immunofluorescence magnification was 630 X, and the levels of alpha-tubulin and beta-actin were calculated by mean ± standard deviation in 3 independent experiments. * represents *p* < 0.05

Because a depletion of *E2F1* changed the cell morphology, the expression of cytoskeletal proteins was tested. The expression levels of α-tubulin and β-actin were examined in H446 and H1688 cells by western blot and immunofluorescence. When *E2F1* was knocked down in H446 cells, α-tubulin and β-actin were decreased (Fig. 3a and b). The same results were observed in H1688 cells when *E2F1* was silenced by *E2F1* specific siRNA (Fig. 3a and b). These results coincide with those shown in Fig. 2, indicating that E2F1 affects cytoskeletal protein expression in SCLC, further implying that the role of E2F1 is important in the process of EMT.

### Expression of E2F family members when *E2F1* was knocked down in H446 cells and silenced in H1688 cells

**Fig. 4:**
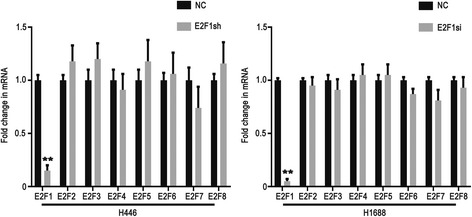
Other E2F family members were detected by real-time PCR when *E2F1* was depleted. The expression levels of other E2F family members were examined by real-time PCR in H446-E2F1sh and H1688-E2F1si cells. The *p* value and error bar was calculated in 3 independent experiments. ** represents *p* < 0.001

The E2F family has 8 members, from *E2F1* to *E2F8*. Although they have similar DNA binding domains, the target genes controlled by E2F family members are different [24]. When *E2F1* was stably knocked down in H446 cells, other E2F family members could have compensated for E2F1 function. We therefore tested the expression of other E2F family members with qPCR in H446 and H1688 cells. The results showed that the *E2F1* expression was significantly knocked down (*p* = 0.0007), and the expressions of *E2F2, E2F3, E2F5* and *E2F8* mRNA was up-regulated, but not to statistically significant levels in H446-E2F1sh cells (Fig. 4). In H1688-E2F1si cells, *E2F1* expression was significantly silenced (*p* = 0.00026) and the mRNA levels of other E2F family members were almost no changed (Fig. 4). These results showed that E2F family members did not compensate for E2F1 when *E2F1* gene was stably knocked out in SCLC cell line.

### E2F1 promoted EMT occurrence in SCLC

**Fig. 5:**
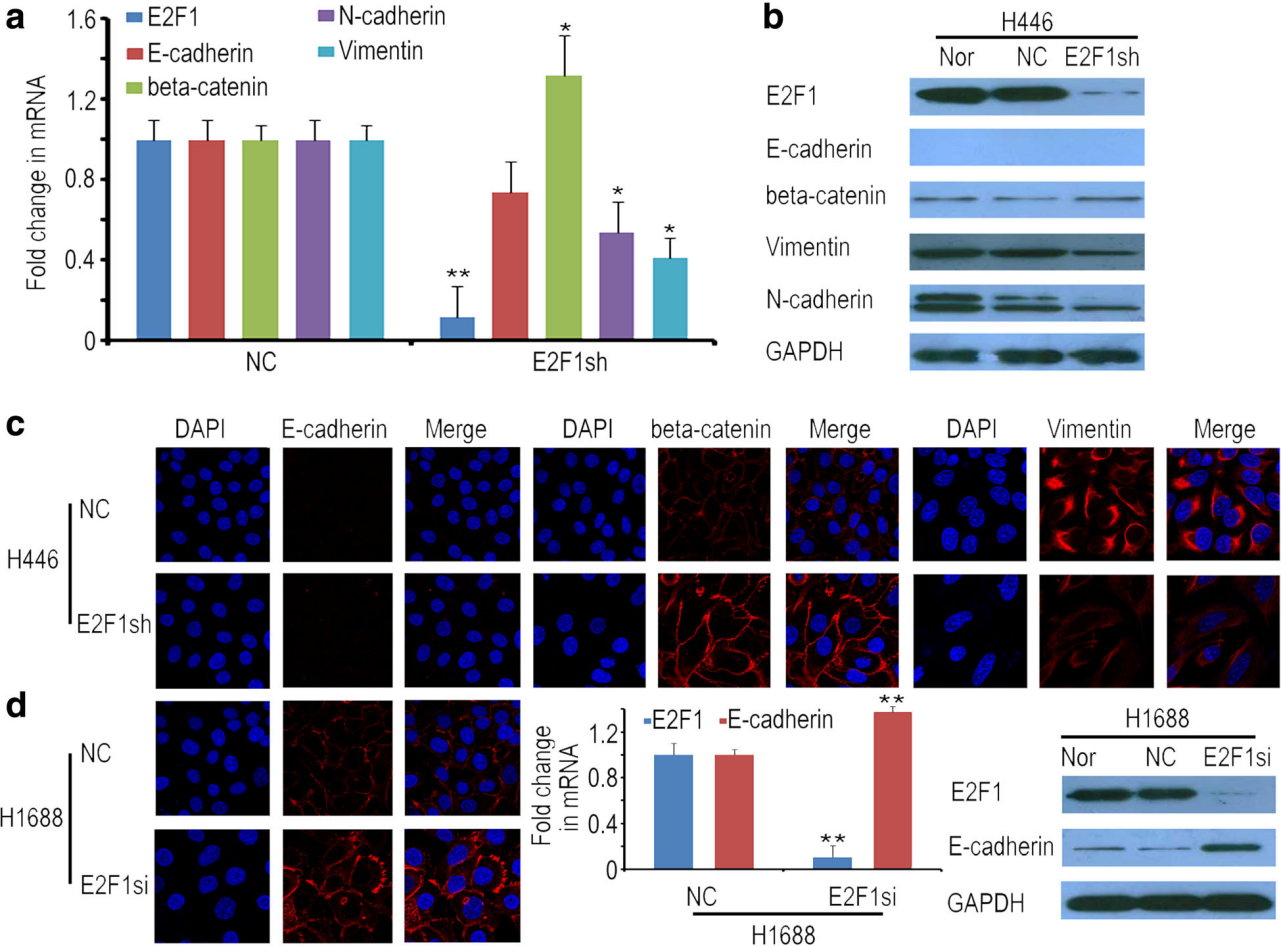
E2F1 influenced the expression of EMT-related proteins. The expression levels of *CDH1, CTNNB1, CDH2* and *VIM* were tested by real-time PCR (**a**), western blotting (**b**) and immunofluorescence (**c**). Because CDH1 protein was not been detected in H446 cells, we selected H1688 to detect the *CDH1* expression (**d**). * represents p < 0.05, ** represents p < 0.001

A depletion of *E2F1* changed the expression of cytoskeletal proteins, and other members of E2F1 family did not compensate E2F1 function. Next, epithelial and mesenchymal markers (CDH1, CTNNB1 and VIM, CDH2) were examined in SCLC cell lines. Real-time PCR showed that *CTNNB1* was significantly increased, while *VIM* and *CDH2* were significantly decreased in H446-E2F1sh cells. Additionally, *CDH1* was decreased, but this change was not statistically significant (Fig. 5a). The protein levels of these EMT markers were consistent with the mRNA levels, with the exception of CDH1 (Fig. 5b, c and Additional file 6: Figure S5A). In H446-E2F1sh cells, we used two different CDH1 antibodies, but CDH1 was not detected by western blot and immunofluorescence. Although CDH1 was highly expressed in SCLC tissue samples (Fig. 1a), it was not present in H446 cells. Therefore, we selected the other SCLC cell line, H1688, to test for *CDH1* expression. When *E2F1* was transiently silenced by siRNAs in H1688 cells, *CDH1* expression was significantly increased at both the mRNA and protein levels (Fig. 5d, Additional file 6: Figure S5B). To further certify that E2F1 could affect the expressions of EMT markers, we selected the lower E2F1 cell line A549 to verify this result. Compared with H1688 and H446, E2F1 expression was lower in A549 cells [2]. Next, *E2F1* was transfected into A549 cells (named A549-E2F1). In A549-E2F1 cells, we found that CDH1 and CTNNB1 were decreased, and VIM and CDH2 were increased in mRNA and protein levels (Additional file 7: Figure S6). These results further indicated that E2F1 could affect the expressions of EMT related markers, and promote EMT occurrence.

### The expression of CDH1 related inhibitory transcription factors in SCLC tissue samples

**Fig. 6:**
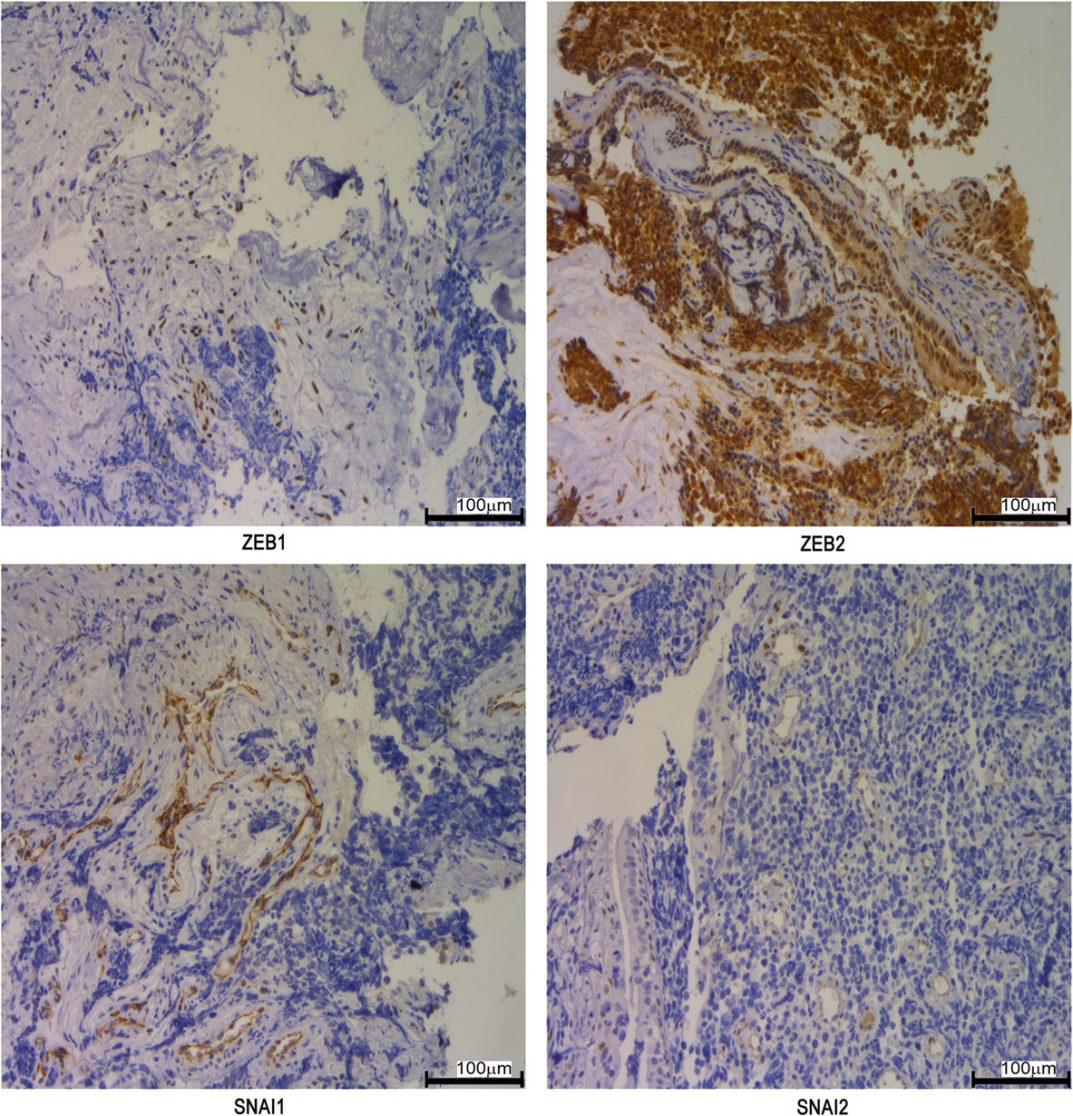
Expressions of ZEB1, ZEB2, SNAI1 and SNAI2 were analysed by IHC in SCLC tissue samples. ZEB1, ZEB2, SNAI1 and SNAI2 were detected in 60 SCLC tissue samples by IHC. Representative image was presented, and the magnification is 200 X

The above results showed that E2F1 promotes EMT, but the mechanism by which it does so is unknown. Previous ChIP-seq studies that examined the target genes of E2F1 in SCLC found that *CDH1, CTNNB1, VIM* and *CDH2* were not directly regulated by E2F1 [2, 25], indicating that E2F1 does not directly control EMT. In addition, ChIP-seq data showed that only *ZEB2* not other known EMT transcription factors was a target of E2F1. We then investigated the expression of *CDH1* inhibitory transcription factors ZEB1, ZEB2, SNAI1 and SNAI2 by immunohistochemistry. The results showed that ZEB2 was the highest among transcriptional repressors of CDH1 (Fig. 6). ZEB2 was found to be strong positive (score between 100 and 300) in 76.67% (46/60) SCLC tissue samples, moderate positive (score between 20 and 100) in 5% (3/60), weak expression (score between 10 and 20) in 6.67% (4/60), and negative expression (score 0) in 11.67% (7/60, Additional file 8: Figure S7). ZEB1 was mainly localized to mesenchymal cells (60/60), not tumour cells (0/60). SNAI1 was mainly localized to the vascular endothelium (55/60). SNAI2 was very weak and only found in a small number of tumour cells (2/60) (Fig. 6). These results were consistent with other reports [15, 18], and indicated that ZEB2 might play an important role in promoting EMT in SCLC.

### E2F1 promoted EMT occurrence in SCLC by regulating *ZEB2* expression

**Fig. 7:**
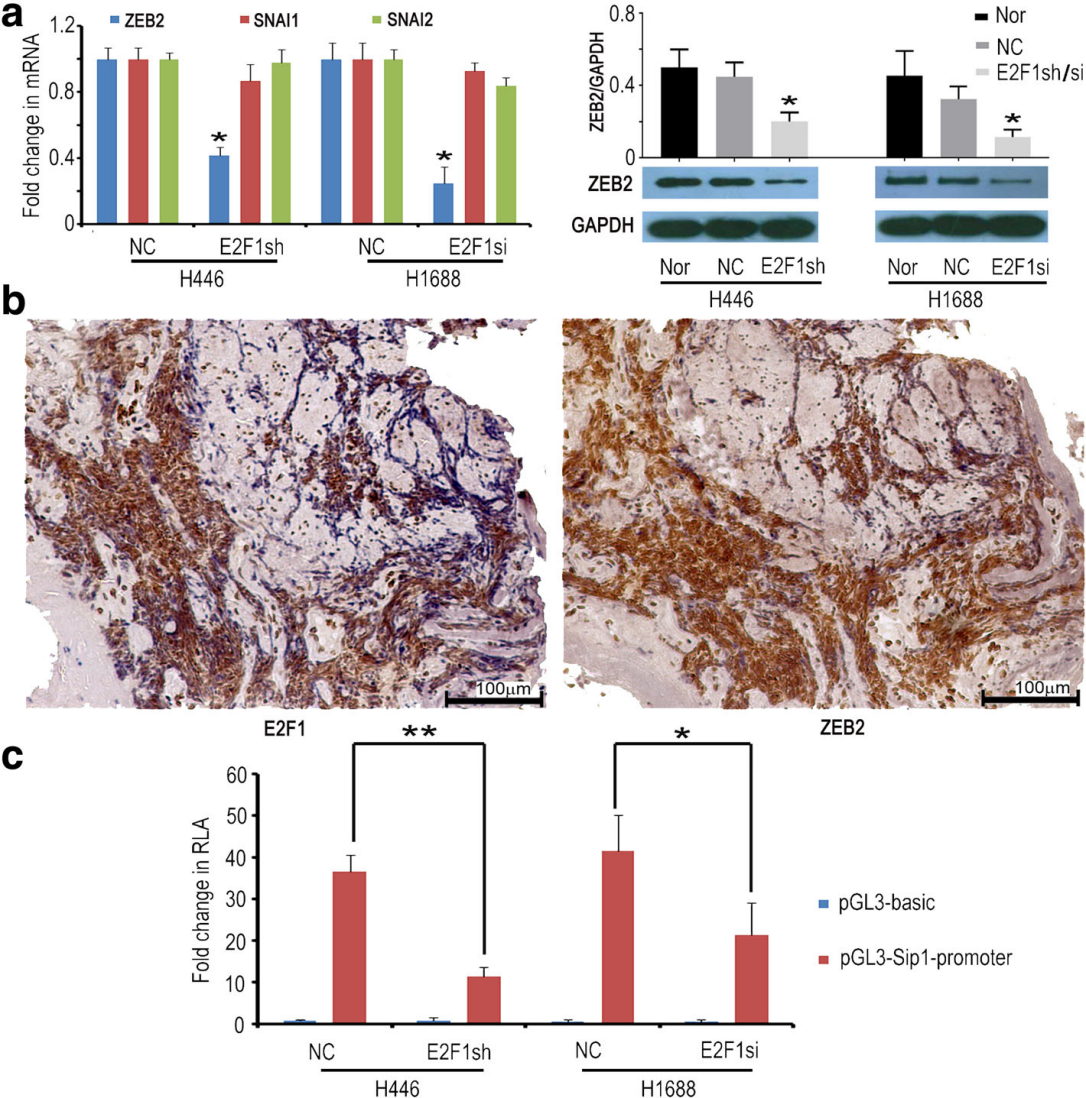
E2F1 could regulate *ZEB2* expression in SCLC cell lines. **a** The expression levels of *ZEB2, SNAI1* and *SNAI2* mRNA were detected by real-time PCR in H446 and H1688 cells. ZEB2 protein was detected in H446-E2F1sh and H1688-E2F1si cells. mRNA levels were calculated in 3 independent experiments. * represents *p* < 0.05. **b** ZEB2 was highly expressed in tissue samples in which E2F1 was also high. Representative image was presented, and the magnification is 200 X. **c** Dual luciferase activity analysis from 3 independent experiments showed that E2F1 could directly regulate *ZEB2* expression in H446 and H1688 cells, * represents p < 0.05, ** represents *p* < 0.001. RLA: Relative luciferase activity

Although ZEB1 and SNAI1 have been found to promote invasion and metastasis in SCLC [26, 27], ZEB1 and SNAI1 was very low in SCLC tissue. We analysed the expression of *ZEB1, ZEB2, SNAI1* and *SNAI2* in H446-E2F1sh and H1688-E2F1si cells by real-time PCR. *ZEB2* mRNA was reduced by 60% and 79%, respectively in H446-E2F1sh and H1688-E2F1si cells, and ZEB2 protein was significantly decreased (Fig. 7a). These results suggest that E2F1 might promote EMT in SCLC by regulating *ZEB2* expression. To test this hypothesis, we found that E2F1 was higher in these SCLC tissues where ZEB2 was strong positive (Fig. 7 b). Our ChIP-seq data (Additional file 9) [2] showed that *ZEB2* was the only target EMT known transcription repressor factor regulated by E2F1. To further certify that E2F1 could regulate *ZEB2* expression in SCLC, we constructed the dual luciferase reporter vectors containing *ZEB2* promoter. After transfection, luciferase activity analysis showed that E2F1 could regulate *ZEB2* expression in H446 and H1688 cells (Fig. 7c). These results further certified our hypothesis that E2F1 promotes EMT by regulating *ZEB2* in SCLC.

## Discussion

E2F1 is a transcription factor that takes part in regulating various biological activities, including the cell cycle [28], apoptosis [29], proliferation [30], angiogenesis [31], tumour drug resistance [32, 33], invasion and metastasis [34, 35] and so on. In our previous studies, we found that E2F1 could regulate MMPs and ADAM-12 to promote invasion and metastasis in SCLC [2, 3]. Recently, several papers have reported that E2F1 can induce EMT in different tumour cells, but the mechanisms described in these studies are inconsistent [13, 36]. In addition, E2F1 was shown to be deregulated upon the loss of Rb in SCLC [37]. Therefore, a role for E2F1 in SCLC has been indicated. Distant metastasis in early stages is a classic biological feature of SCLC, and EMT is considered to be an early, essential step for invasion and metastasis. It is unknown how E2F1 regulates EMT in SCLC. In this study, we detected and analysed the expression of EMT markers in SCLC tissue samples and found that CDH1, CTNNB1 and VIM were closely related with clinical stage and differentiation [38]. When E2F1 was depleted, the cellular morphology changed from grain-shaped to slender and fibrous. Additionally, cytoskeletal proteins underwent remodelling and EMT markers were significantly changed, indicating that E2F1 plays a role in EMT in SCLC. Next, we found that ZEB2 was highly expressed in the same tissues in which E2F1 was highly expressed. ChIP-seq data and luciferase analysis indicated that E2F1 could control *ZEB2* expression in SCLC.

EMT is a process whereby epithelial cell polarity is lost, cellular adhesions are weakened, and the cytoskeleton is remodelled [10]. Tumour cells are surrounded by mesenchymal cells. For tumour cells to move a distance, they must break through the surrounding mesenchymal cells. To do this, they have to disguise themselves as mesenchymal cells. EMT is considered to be the first step in breaking through the mesenchymal cell defence. A loss of the CDH1/CTNNB1 complex is a marker of EMT occurrence. This complex plays an important role in mediating intercellular adherence junction and maintaining epithelial integrity [39]. A “Cadherin Switch” refers to the change from CDH1 to CDH2 and is considered to be the key factor in tumour invasion and metastasis [40]. The expressions of CDH1 and CTNNB1 were significantly weaker in tumour tissue than normal bronchial epithelial cells [41]. In our study, CDH2 was not detected in SCLC tissue samples, but was decreased in H446-E2F1sh cells. In H1688 cells, when E2F1 was silenced, CDH1 was increased and CDH2 was decreased. In addition, CDH1 expression was decreased and CDH2 expression was increased in A549-E2F1 cells. A previous study showed that ZEB2 played an important role in the “Cadherin Switch” during cranial neural crest EMT [42]. In our study, we found that ZEB2 was regulated by E2F1 in SCLC, and we speculate that E2F1 might drive the “Cadherin Switch” to further promote EMT in SCLC.

Interestingly, some papers reported that EMT rarely occurs homogenously across the whole tumour, and it is hypothesized that EMT is transient and occurs at the tumour margin tissues [23, 43, 44]. In our study, we found that VIM is higher in adjacent mesenchymal tumour cells, and is very lower in the tumour central sites. This result further confirmed this conclusion that EMT is transient and occurs at the tumour margin tissues. The same results were also observed in non-small cell lung cancer (NSCLC). Mahmood MQ et al. found that VIM and CDH2 were higher in tumour cells located at the peripheral leading edge of NSCLC when compared with centrally located tumour cells of same subjects [45].

*ZEB2* is expressed in various human tumours, including liver cancer [46], colorectal cancer [47] and breast cancer [48]. Our results showed that ZEB2 was higher than ZEB1, SNAI1 and SNAI2 in SCLC tissue samples, which is inconstant with other reports [49, 50]. Additionally, research on ZEB2 as an EMT facilitator has been focused on because of its role as a transcriptional repressor of CDH1 [51], with little research being done on the regulation of *ZEB2* expression. SNAI11 could increase *ZEB2* expression at the translational level, as opposed to the transcriptional level, by inhibiting the splicing of the 5′-UTR in the *ZEB2* intron [52]. Integrative genomic analyses showed that SMAD, ETS1, HIF1α, POU/OCT and NF-κB could affect *ZEB2* transcription [53]. In our study, we found that *E2F1* expression was highly consistent with *ZEB2* expression. ChIP-seq data and luciferase activity also showed that E2F1 regulated *ZEB2* expression through E2F1 binding sites on the promoter of *ZEB2* in SCLC.

In summary, our data supports the idea that E2F1 promotes EMT by regulating *ZEB2* expression in SCLC.

## Conclusion

Transcript factor E2F1 promotes EMT by regulating *ZEB2* gene expression, and then participates in invasion and metastasis of SCLC.

## Additional files

Additional file 1: The specific primers for target genes. (DOCX 19 kb)

Additional file 2: Figure S1. The differential expression intensity of CDH1 and CTNNB1 in SCLC tissue samples. (TIFF 10260 kb)

Additional file 3: Figure S1. The staining of CDH1 and CTNNB1 in bronchial epithelial cells. (TIFF 6069 kb)

Additional file 4: Figure S3. The expression levels of *CDH1* and *CTNNB1* in differential SCLC cell lines. (TIFF 13906 kb)

Additional file 5: Figure S4. *E2F1* expression was quantified in H446-E2F1sh and H1688-E2F1si cells. * represents *p* < 0.05, ** represents *p* < 0.001. (TIFF 3949 kb)

Additional file 6: Figure S5. The relative protein levels of E2F1, CTNNB1, VIM and CDH2 in H446-E2F1sh cells, and E2F1, CTNNB1 in H1688-E2F1si cells. (TIFF 533 kb)

Additional file 7: Figure S6. E2F1 overexpression in A549 cells could inhibit the expression of *CDH1* and *CTNNB1*, and promote the expression of *CDH2*. (TIFF 6963 kb)

Additional file 8: Figure S7. The differential expression intensity of ZEB2 in SCLC tissue samples. (TIFF 7166 kb)

Additional file 9: The target genes of E2F1 by ChIP-seq in H1688 cell line. (XLSX 431 kb)

## Abbreviations

ADAM: A disintegrin and metalloprotease
ChIP-seq: Chromosome immunoprecipitation to sequencing
EMT: Epithelial-mesenchymal transition
MMPs: matrix metalloproteinases
PDGFR: platelet-derived growth factor receptor
SCLC: small cell lung cancer
VEGFR: vascular endothelial growth factor receptor
