# Supplementary material for: Transcription factor E2F1 promotes EMT by regulating ZEB2 in small cell lung cancer
Source: BMC Cancer. 2017 Nov 7;17:719. doi: 10.1186/s12885-017-3701-y (PMC5678576; doi:10.1186/s12885-017-3701-y)
Supplement: Supplementary file 1 — The specific primers for target genes. (DOCX 19 kb) [file 12885_2017_3701_MOESM1_ESM.docx]

Additional file 1 Table S1 Specific primers for target genes

| Target gene | Primers |
| --- | --- |
| E2F1 | F: 5’-CATCAGTACCTGGCCGAGAG-3’ |
|  | R: 5’-TGGTGGTCAGATTCAGTGAGG-3’ |
| E2F2 | F: 5’-CGTCCCTGAGTTCCCAACC-3’ |
|  | R: 5’-GCGAAGTGTCATACCGAGTCTT-3’ |
| E2F3 | F: 5’-GTATGATACGTCTCTTGGTCTGC-3’ |
|  | R: 5’-CAAATCCAATACCCCATCGGG-3’ |
| E2F4 | F: 5’-CACCACCAAGTTCGTGTCCC-3’ |
|  | R: 5’-GCGTACAGCTAGGGTGTCA-3’ |
| E2F5 | F: 5’-TCATTCAGGACCTATCCATGTGC-3’ |
|  | R: 5’-GTCACTGGAGTCAAGGACTGG-3’ |
| E2F6 | F: 5’-TCCATGAACAGATCGTCATTGC-3’ |
|  | R: 5’-TCCGTTGGTGCTCCTTATGTG-3’ |
| E2F7 | F: 5’-TAGCTCGCTATCCAAGTTATCCCT-3’ |
|  | R: 5’-CAATGTCATAGATGCGTCTCCTT-3’ |
| E2F8 | F: 5’-AAGTACGCCGAGCAGATTATG-3’ |
|  | R: 5’-ATGTCTGGGTGTCCATTTGGG-3’ |
| CDH1 | F: 5’-CCCACCACGTACAAGGGTC-3’ |
|  | R: 5’-CTGGGGTATTGGGGGCATC-3’ |
| CTNNB1 | F: 5’-GTCGAGGACGGTCGGACT-3’ |
|  | R: 5’-TGTCCACGCTGGATTTTCAA-3’ |
| CDH2 | F: 5’-CGAATGGATGAAAGACCCATCC-3’ |
|  | R: 5’-GGAGCCACTGCCTTCATAGTCAA-3’ |
| VIM | F: 5’-AGTCCACTGAGTACCGGAGAC-3’ |
|  | R: 5’-CATTTCACGCATCTGGCGTTC-3’ |
| ZEB2 | F: 5’-GCCTCTGTAGATGGTCCAGTGA-3’ |
|  | R: 5’- TCACTGCGCTGAAGGTACTC-3’ |
| SNAI1 | F: 5’-AGCGAGCTGCAGGACTCTAA-3’ |
|  | R: 5’- ATCTCCGGAGGTGGGATGG-3’ |
| SNAI2 | F: 5’-CAACGCCTCCAAAAAGCCAA -3’ |
|  | R: 5’-TCTGGTTGTGGTATGACAGGC -3’ |
| 18sRNA | F: 5’-ACATCCAAGGAAGGCAGCAG-3’ |
|  | R: 5’-TTCGTCACTACCTCCCCGG-3’ |
| ZEB2 promoter | F: 5’-ACT *GCTAGC* CATTGGTTAAGAACACTTAAACTT -3’ |
|  | R: 5’-ACT *AGATCT*CTTTTCTGTAGGAGAGAGACGA -3’ |
