# Supplementary figures and images for: Transcription factor E2F1 promotes EMT by regulating ZEB2 in small cell lung cancer
Source: BMC Cancer. 2017 Nov 7;17:719. doi: 10.1186/s12885-017-3701-y (PMC5678576; doi:10.1186/s12885-017-3701-y)

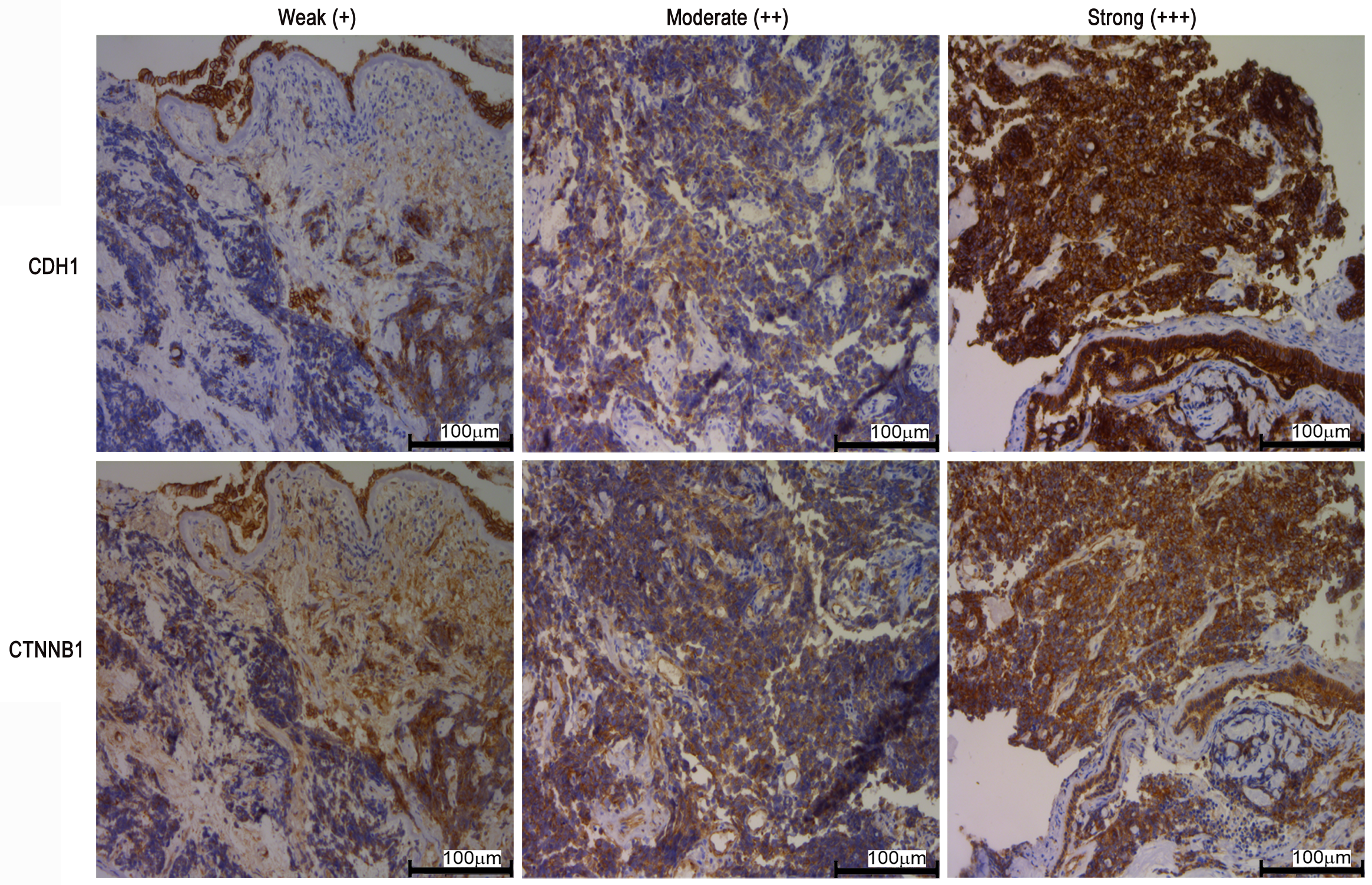

Supplement: Supplementary file 2 — The differential expression intensity of CDH1 and CTNNB1 in SCLC tissue samples. (TIFF 10260 kb) [file 12885_2017_3701_MOESM2_ESM.tif]

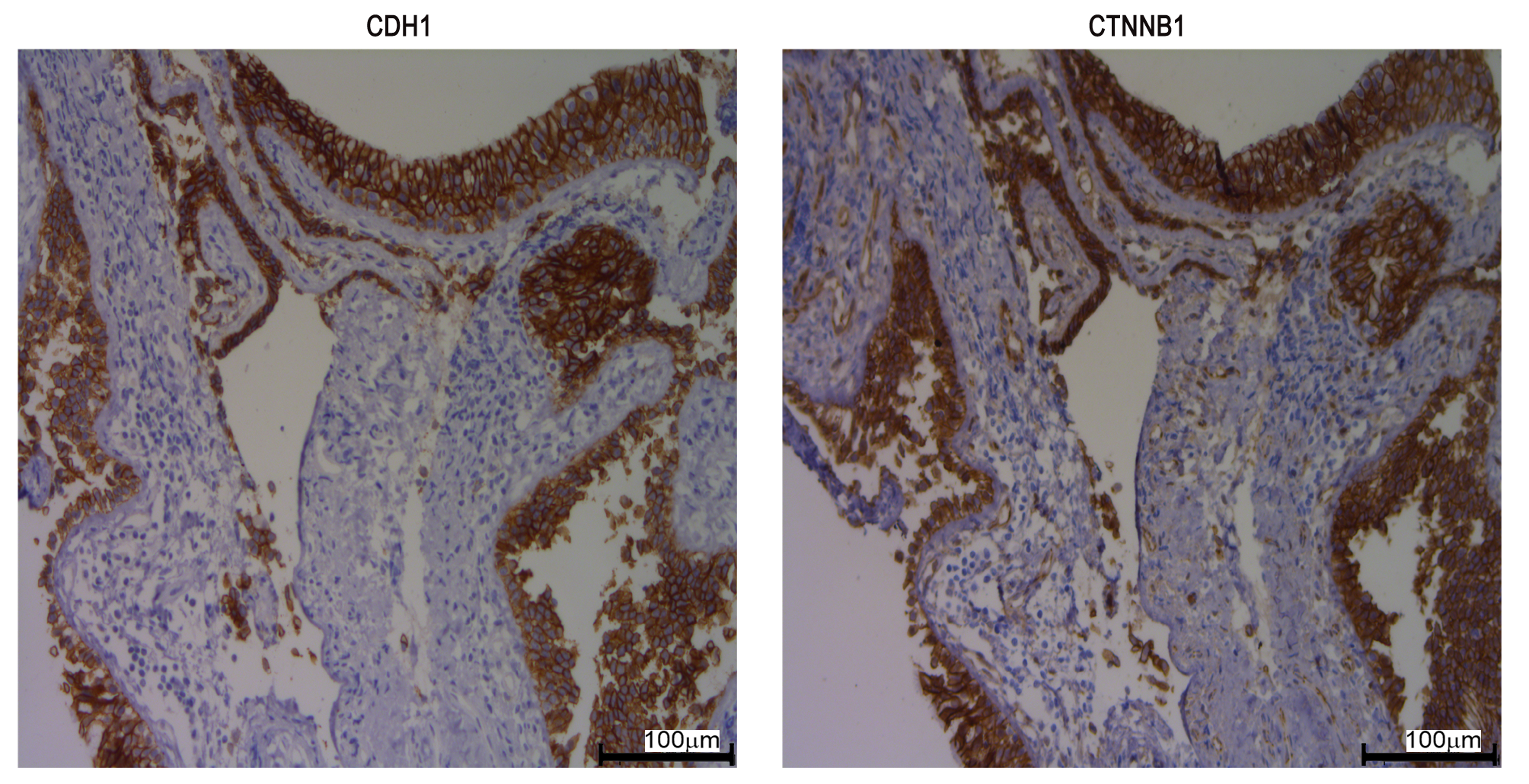

Supplement: Supplementary file 3 — The staining of CDH1 and CTNNB1 in bronchial epithelial cells. (TIFF 6069 kb) [file 12885_2017_3701_MOESM3_ESM.tif]

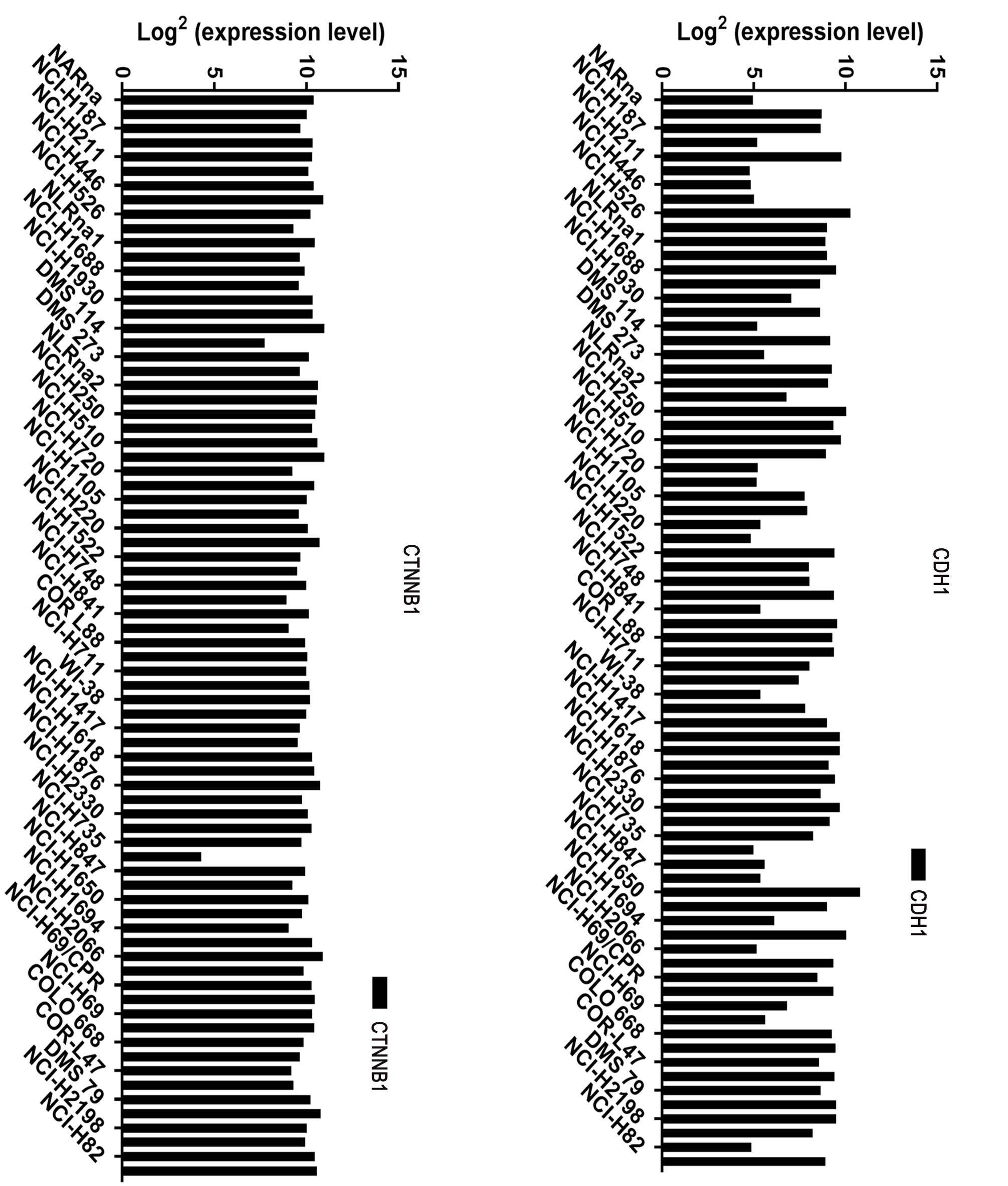

Supplement: Supplementary file 4 — The expression levels of CDH1 and CTNNB1 in differential SCLC cell lines. (TIFF 13906 kb) [file 12885_2017_3701_MOESM4_ESM.tif]

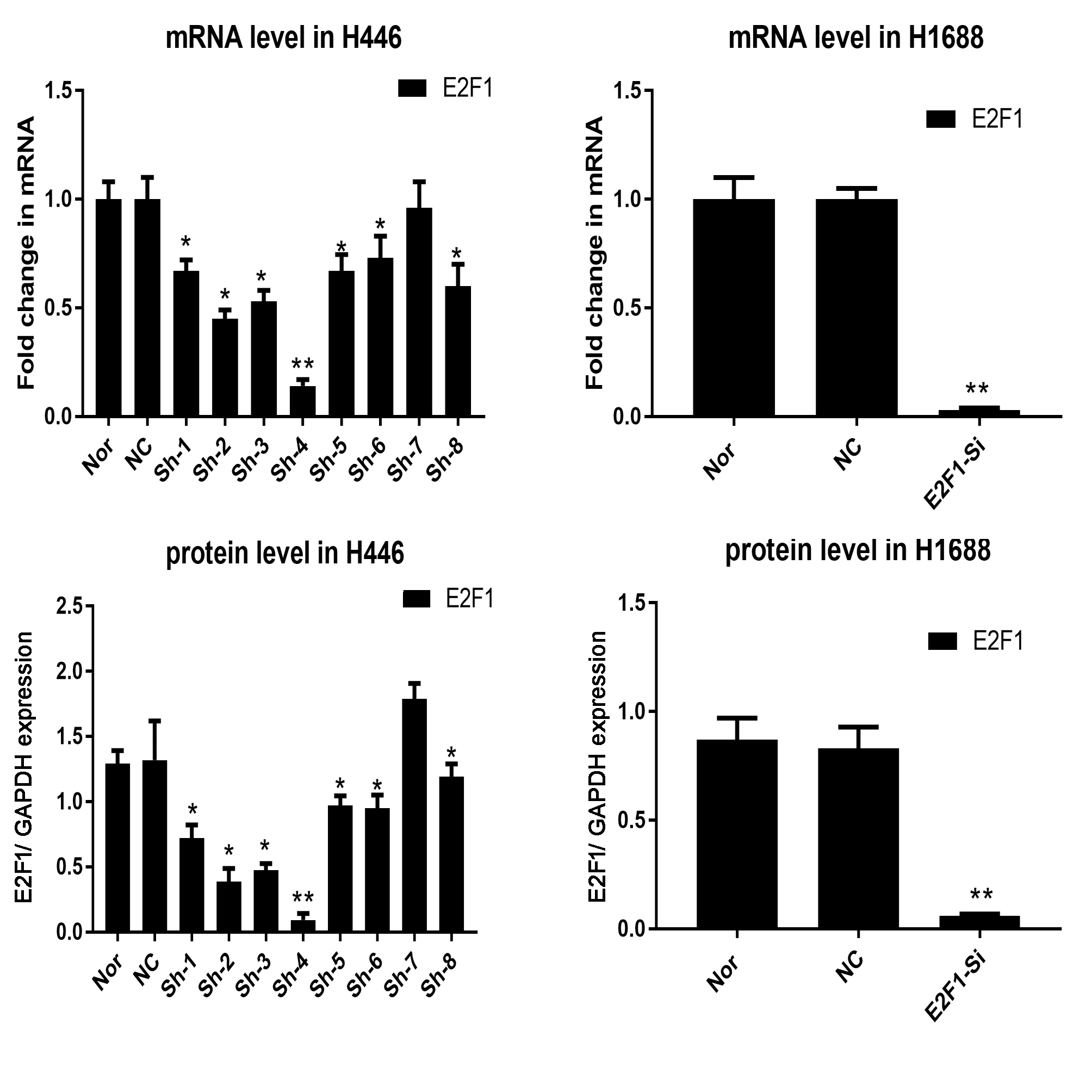

Supplement: Supplementary file 5 — E2F1 expression was quantified in H446-E2F1sh and H1688-E2F1si cells. * represents p < 0.05, ** represents p < 0.001. (TIFF 3949 kb) [file 12885_2017_3701_MOESM5_ESM.tif]

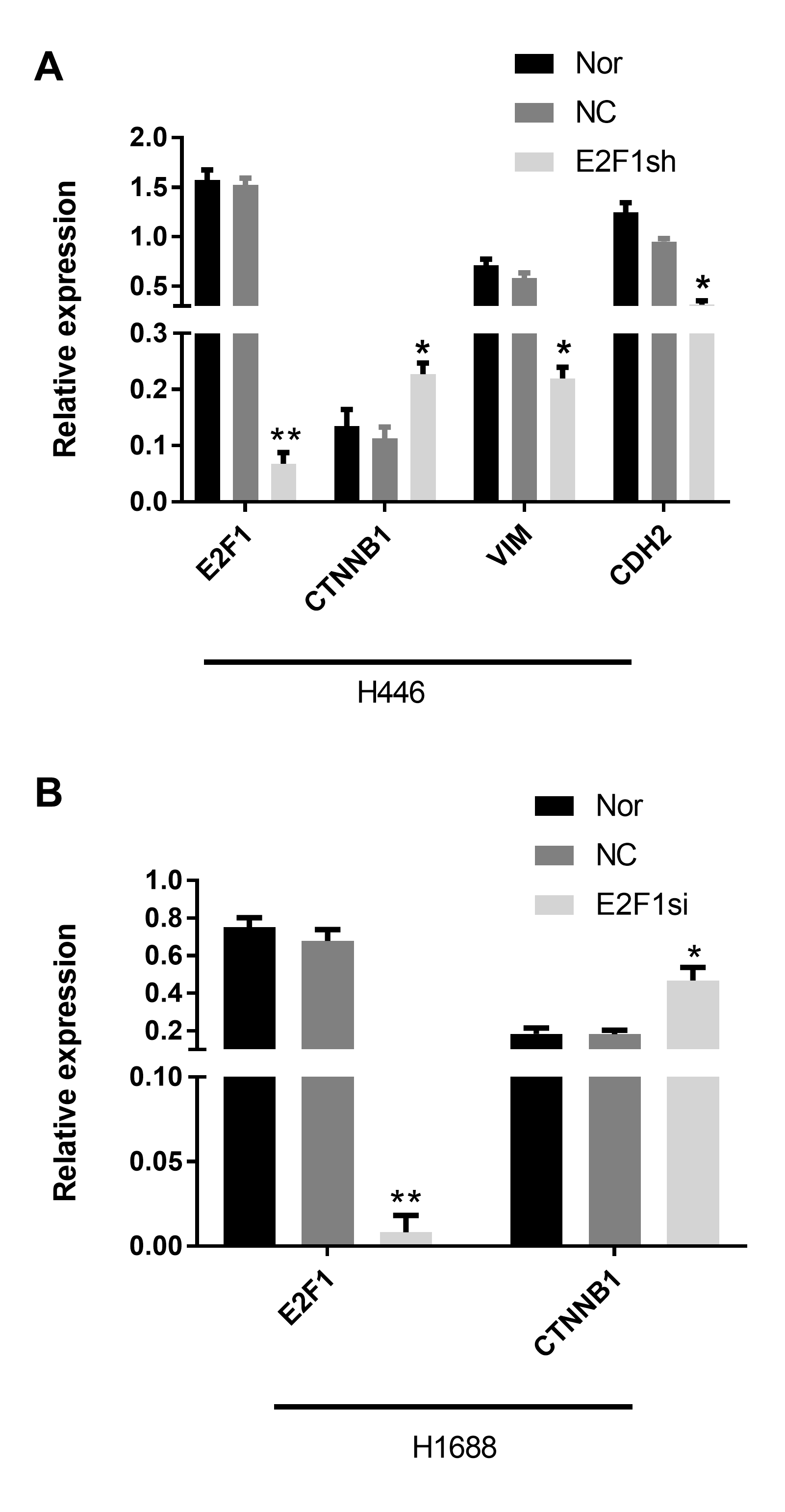

Supplement: Supplementary file 6 — The relative protein levels of E2F1, CTNNB1, VIM and CDH2 in H446-E2F1sh cells, and E2F1, CTNNB1 in H1688-E2F1si cells. (TIFF 533 kb) [file 12885_2017_3701_MOESM6_ESM.tif]

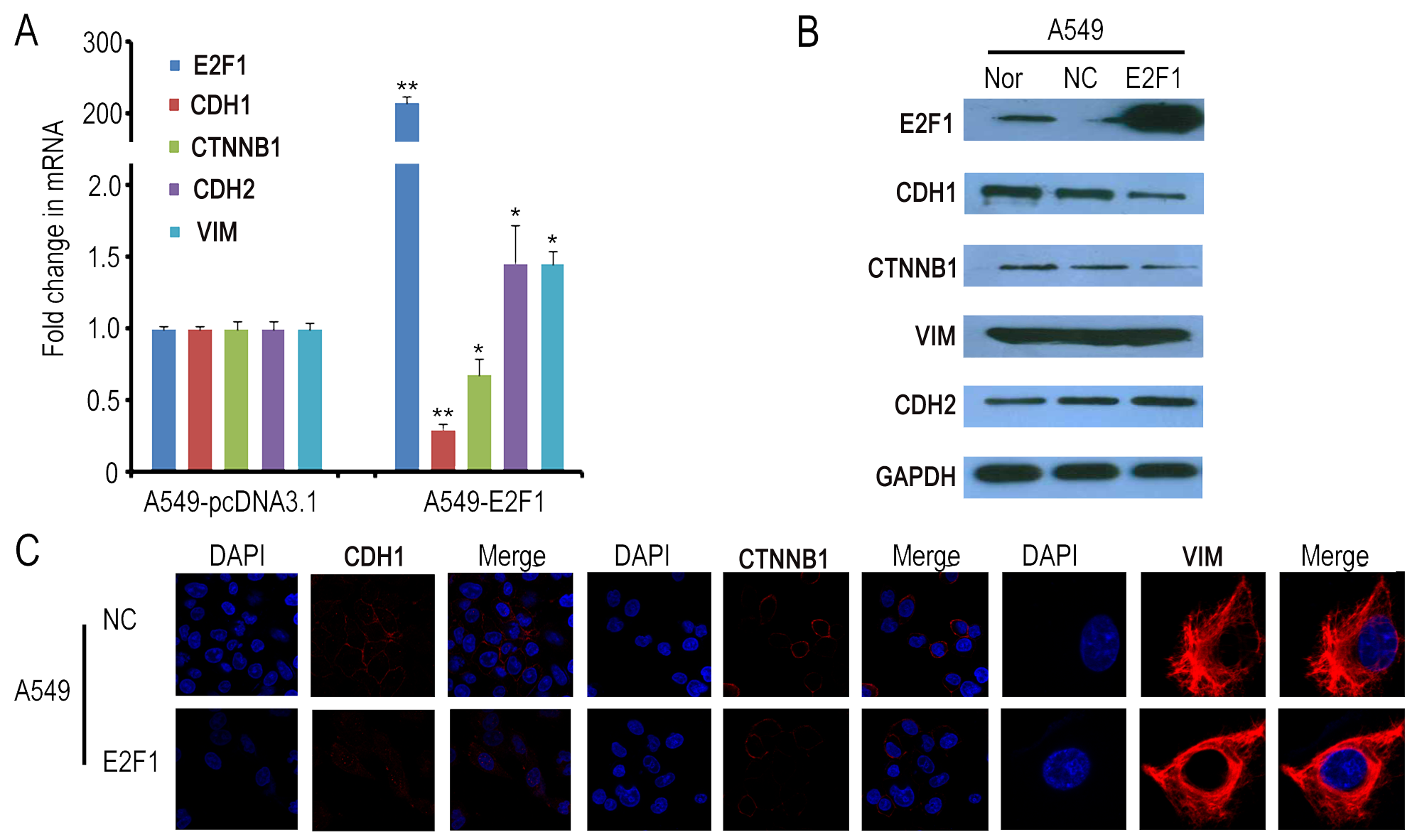

Supplement: Supplementary file 7 — E2F1 overexpression in A549 cells could inhibit the expression of CDH1 and CTNNB1, and promote the expression of CDH2. (TIFF 6963 kb) [file 12885_2017_3701_MOESM7_ESM.tif]

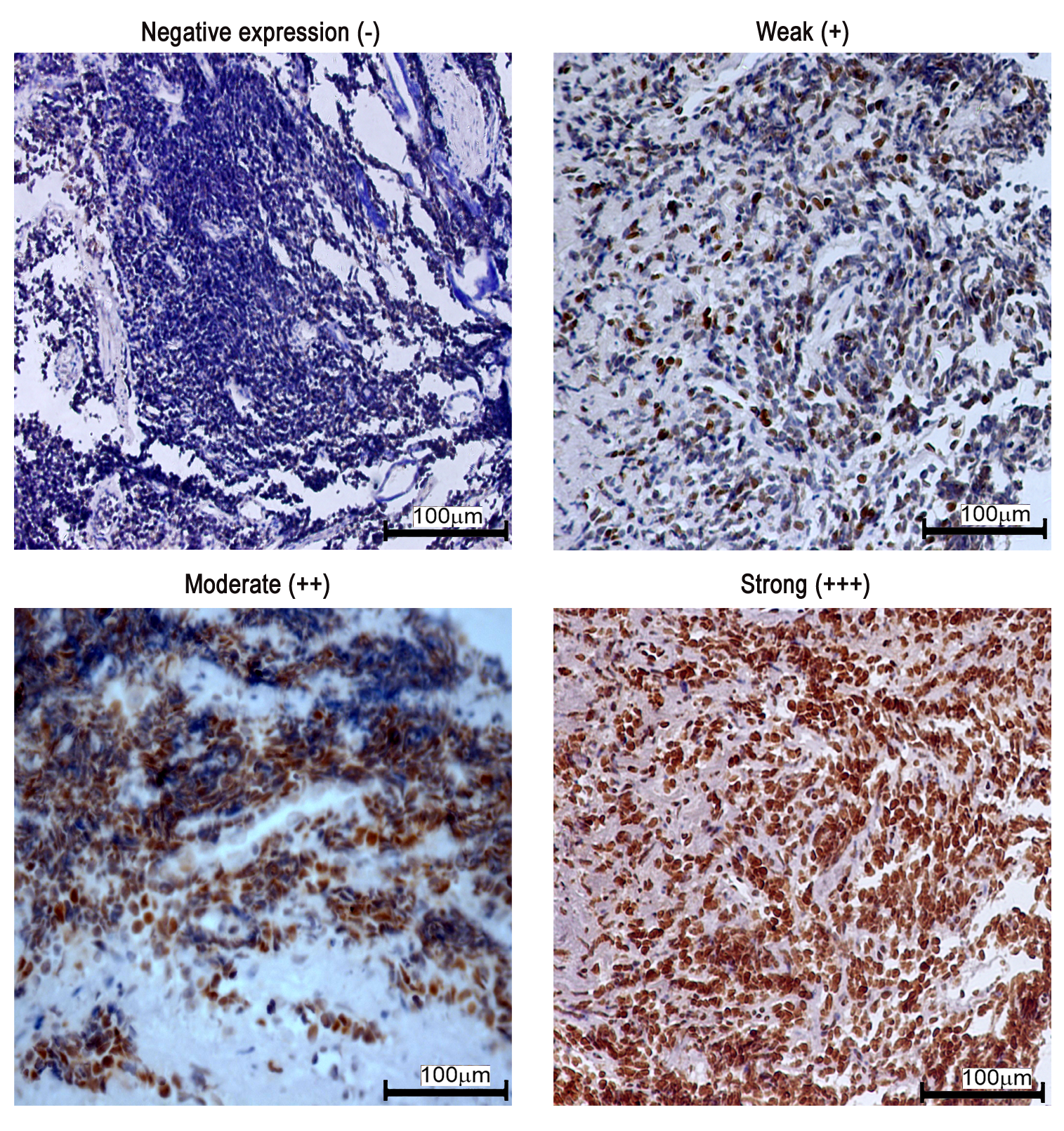

Supplement: Supplementary file 8 — The differential expression intensity of ZEB2 in SCLC tissue samples. (TIFF 7166 kb) [file 12885_2017_3701_MOESM8_ESM.tif]
